# Paradoxical Roles of Tumour Necrosis Factor-Alpha in Prostate Cancer Biology

**DOI:** 10.1155/2012/128965

**Published:** 2012-12-27

**Authors:** Brian W. C. Tse, Kieran F. Scott, Pamela J. Russell

**Affiliations:** ^1^Australian Prostate Cancer Research Centre-Queensland, Queensland University of Technology, Brisbane, QLD 4102, Australia; ^2^Institute of Health and Biomedical Innovation, Cells and Tissue Domain, Faculty of Health, Queensland University of Technology, Brisbane, QLD 4059, Australia; ^3^Department of Medicine, St. George Hospital Clinical School, The University of New South Wales, Sydney, NSW 2217, Australia

## Abstract

Tumour necrosis factor (TNF) is a pleiotropic cytokine with dual roles in cancer biology including prostate cancer (PCa). On the one hand, there is evidence that it stimulates tumour angiogenesis, is involved in the initiation of PCa from an androgen-dependent to a castrate resistant state, plays a role in epithelial to mesenchymal plasticity, and may contribute to the aberrant regulation of eicosanoid pathways. On the other hand, TNF has also been reported to inhibit neovascularisation, induce apoptosis of PCa cells, and stimulate antitumour immunity. Much of the confusion surrounding its seemingly paradoxical roles in cancer biology stems from the dependence of its effects on the biological model within which TNF is investigated. This paper will address some of these issues and also discuss the therapeutic implications.

## 1. Introduction

Cytokines are soluble, low molecular weight proteins that mediate cell-cell communication. They are mainly produced by immune cells and stromal cells (including fibroblasts and endothelial cells) and act in concert to regulate biological responses such as cell activation, proliferation, differentiation, migration, and cytotoxicity. Cytokines have an integral role in tumour induction and progression. They can facilitate the generation and maintenance of robust antitumour immune responses, but they can also contribute to chronic inflammation and promote tumour formation, growth and metastasis [1]. Whether the cytokine network within a given tumour microenvironment is conducive or inhibitory for tumour growth is highly dependent on the array of the cytokines present, their relative concentrations, cytokine receptor expression patterns, and the activation status of cells that express these receptors. One cytokine that exhibits dual roles in tumourigenesis is tumour necrosis factor-alpha (TNF-*α*; also referred to as TNF). True to its name, TNF is cytotoxic to tumour cells under certain conditions; however, it also fuels tumour-promoting inflammation and angiogenesis. This paper will discuss the function of TNF in cancer biology, with special emphasis on PCa.

## 2. TNF Signalling

TNF is a multifunctional cytokine first isolated from the serum of Bacillus Calmette-Guerin- (BCG-)infected mice treated with endotoxin that could induce hemorrhagic necrosis of tumours in mice [2]. It is synthesized as a 26 kD membrane-bound protein and cleaved into a 17 kD soluble protein by TNF-converting enzyme (TACE) [3]. TNF is predominantly produced by macrophages, T cells and natural killer (NK) cells, but nonimmune cells such as fibroblasts, smooth muscle cells, and tumour cells have also been reported to secrete low amounts of the cytokine [4]. TNF signals via two distinct receptors: TNF receptor-1 (TNFR-1, p55 receptor), which is ubiquitously expressed, and TNF receptor-2 (TNFR-2, p75 receptor), which is mainly expressed on immune cells. TNFR-1 transduces both proapoptotic as well as prosurvival signals, although the mechanisms that regulate the life or death outcome are not well understood [4]. Upon binding to TNF, TNFR-1 trimerises, causing the silencer of death domain (SODD) protein to be released from the DD of TNFR-1 [5]. This permits the assembly of a complex composed of TNFR-associated death domain (TRADD), TNFR-associated factor 2 (TRAF2), receptor-interacting protein (RIP), and FAS-associated death domain (FADD) (together called complex 1) [5]. When TNFR-1 signals cell death, FADD binds to procaspase-8, which triggers the activation of other caspases and endonucleases that result in DNA fragmentation and destruction of intracellular proteins, and eventually, apoptosis [4]. In another apoptosis-inducing pathway, TRAF2 activates the cascade signal regulating kinase (ASK-1), mitogen activated protein kinase-kinase 4 (MEK4), and Jun N-terminal kinase (JNK), which then phosphorylates activator protein-1 (AP-1), stimulating apoptosis [6]. When TNFR-1 signals survival, inhibitor of *κ*B (I*κ*B) kinase (IKK) is recruited to complex 1 and is activated via RIP-dependent mechanisms. The activated IKK phosphorylates I*κ*B triggers its ubiquitination then degradation in the proteasome; hence allowing nuclear-factor- (NF-)*κ*B to translocate from the cytoplasm to the nucleus to promote transcription of target antiapoptotic genes such as B-cell lymphoma extra-large (Bcl-xL), A20, cellular inhibitor of apoptosis protein (cIAP-)1 and 2 [5, 7]. Another antiapoptotic pathway is triggered by the binding of TRAF2 with cIAP-1 [3]. TNFR-2 signaling mainly occurs on immune cells and endothelial cells, and less is known about its mechanisms of signal transduction. However, TNFR-2 lacks a death domain, but can affect NF-*κ*B and JNK signaling [3].

## 3. TNF as a Biomarker of PCa

An ideal cancer biomarker is one that allows for early detection of disease and/or assessment of response to therapy and prognosis, is minimally invasive to the patient when sampled, and cost effective to be assayed [8]. Since cytokines are often involved in the evolution of cancers, measuring their levels in bodily fluids may provide a reflection of the patient's pathological state. Serum TNF levels have been shown to be reflective of tumour load in PCa patients being low in healthy men (mean 1.1 ± 0.5 pg/mL), higher in patients with bulky locally-advanced PCa (3.9 ± 3.4 pg/mL), and highest in those with metastatic disease (lymph node and bone involvement) (6.3 ± 3.6 pg/mL) [9]. When patients develop symptomatic progressive disease, serum TNF levels also significantly elevate from initial presentation. A univariate analysis of serum TNF levels in relation to survival showed that patients with locally-advanced PCa and high TNF (>1.9 pg/mL; cut-off defined as the 95th percentile of values in control group) had significantly shorter survival as compared to their counterparts with low TNF (*P* = 0.04) [9]. As PCa patients often experience cachexia (weight loss, anorexia, anaemia, and metabolic abnormalities), a study evaluated the relationship between this disease complication and serum TNF levels. Patients with elevated serum TNF (defined as >2 units/mL) have lower levels of serum albumin and hemoglobin, lower body mass index, and shorter survival time [10]. Similarly, high serum TNF correlates with increases in plasma levels of thrombin-antithrombin-III complex, plasmin-*α*2-antiplasmin inhibitor complex, and soluble fibrin monomer complex, hence, linking TNF with coagulopathy in PCa [11]. While it cannot be ascertained from these studies whether the elevated TNF contributes to disease progression, or is a reflection of advanced disease, it is clear that TNF is a potential PCa biomarker, and more research into its clinical diagnostic and prognostic utility is warranted.

In prostatic tissues, TNF expression levels correlate with disease progression, with immunostaining for TNF reported to be absent on normal prostatic (NP) epithelial cells, weak on benign prostatic hyperplasic (BPH) tissues but strong on prostatic carcinomas [12]. Similarly, both BHP and PCa express significantly higher levels of TNFR-1 and TNFR-2 as compared to the NP tissue [13]. This observation is consistent with findings from a recent study which analysed genome wide methylation in PCa [14]. The *TNFRSF1B* gene, which encodes TNFR-2, was hypo-methylated by 3-fold in PCa samples, whilst the two genes, *BCL-2* and *BAK1*, which are involved in TNF-dependent apoptosis pathways, were found to be hyper-methylated, resulting in their downregulation [14]. Evidence for alterations in TNF-mediated apoptosis pathways in PCa was also provided by another study where immunostaining for TRAF-2, ASK-1, MEK-4 and JNK (involved in proapoptotic pathway) was intense on biopsies from normal prostates, weaker in BHP but absent in PCas, which may in part account for resistance to TNF-mediated death by in PCa [6]. On the other hand, immunostaining for NF-*κ*B inducing kinase (NIK), IKK, I*κ*B*α*, p-I*κβ*, p50, and p65 (involved in TNF-mediated prosurvival pathway) was progressively elevated from NP to BHP, and to PCa [15].

## 4. Role of TNF in Initiation and Progression of PCa to a Hormone-Refractory State

PCa is regulated by androgen dependent gene pathways. Despite effective surgery or radiation therapy, over 25% of patients will suffer a relapse and face hormone deprivation therapy (ADT) which improves the time to clinical progression and symptom management [16]. ADT effectiveness is due to the requirement for androgens by the prostate gland and PCa for growth and survival [17]. Androgen removal thus initially induces tumour regression and a period of cancer control. Therapeutic approaches to ADT have evolved from surgical castration to safer, direct approaches that interfere with the hypothalamic gonadal axis for testosterone synthesis [18]. The past 3 decades have seen additional and sequential use of androgen receptor (AR) antagonists that bind directly to the AR to inhibit its action [19]. The AR is a ligand regulated receptor; upon binding the physiological androgen, dihydrotestosterone (DHT), the AR serves to activate and repress a large set of responsive genes that control growth, stress, proliferation, differentiation, and cell survival. While these therapies are highly effective and lead to remissions typically lasting 2-3 years, all patients will eventually develop castrate resistant prostate cancer (CRPC), which has no cure [20]. Treatment relapse is partly due to the ability of CRPC to undertake *de novo* steroidogenesis and synthesis of androgens and other steroids that reactivate the AR [21]. Emerging evidence indicates that TNF has key roles in both castration-induced regression of the normal prostate, as well as in PCa progression to a castrate resistant state. A recent study showed that after surgical castration of mice, the prostates from TNF^−/−^ mice regressed significantly more slowly than those from wild-type mice, and that regression could be restored following administration of soluble TNF [22]. The slower rate of castration-induced prostate regression was also observed in TNFRI^−/−^ mice, suggesting that TNF death signalling is required for normal prostate regression [22]. In addition, the authors demonstrated that membrane-bound TNF increased by 2-fold in the ventral prostate of rats following castration, and that this was paralleled with a 50–500-fold increase in mRNA level of TNF within the stromal compartment of the ventral prostate, suggesting that the stroma could be a rich source of regression-mediating TNF. However, studies using the androgen sensitive cell line, LNCaP, also provide evidence that TNF may be involved in the progression of PCa to a castrate resistant state. TNF was shown to dose-dependently decrease the expression of the androgen receptor (AR) and inhibit dihydrotestosterone (DHT)-induced proliferation of LNCaP cells, suggesting that TNF may play a role in the initiation of an androgen-independent state in these cells [12]. In LN-TR2 cells, a subline of LNCaP cells derived from long term culture in low levels of TNF, maximal DHT-induced cell proliferation was achieved with 10-fold less DHT as compared to that required for the parental cells [23]. In addition, LN-TR2 cells showed higher expression of nuclear AR as well as the AR coactivators, androgen receptor associated protein-55 (ARA55), and transcriptional intermediary factor-2 (TIF2), which correlated with enhanced transcriptional activity of AR and prostate specific antigen (PSA) [23, 24]. These results indicate that chronic exposure to low amounts of TNF induces hypersensitivity to androgen in LNCaPs; and this mechanism could play a role in hormone-resistance, at least in some patients with PCa.

## 5. TNF Acts as a Double-Edged Sword in Tumour Progression

TNF exhibits both tumour-promoting and tumour-inhibitory properties, depending on the experimental context within which the conclusions are made (Table 1). There is evidence that chronic synthesis of low amounts of TNF within a tumour microenvironment promotes tumour growth and favours angiogenesis, whereas higher doses can induce necrosis of tumour cells, stimulate antitumour immunity, and trigger vascular collapse [25, 26]. It is now accepted that chronic inflammation is a major risk factor for carcinogenesis, and emerging evidence shows that TNF has key roles in this process [26]. In a *de novo* carcinogenesis model in which the carcinogens 7,12-dimethylbenz[*α*]anthracene (DMBA) and 12-O-tetradecanoyl-phorbol-13-acetate (TPA) were applied to the skin of mice, TNF^−/−^ mice were significantly more resistant to tumour induction than wild-type animals [27]. TNF^−/−^ mice had a lower incidence of total tumours, and where these did develop, there was a delay in onset as compared to those in wild-type mice. Histologically, tumours from wild-type mice contained a heavy infiltrate of inflammatory neutrophils and eosinophils, whereas only a mild infiltrate was seen in tumours from TNF^−/−^ mice [27]. In addition, the skin of TNF^−/−^ mice contained lower levels of myeloperoxidase (MPO), a constituent of neutrophil granules which contribute to carcinogenesis via the generation of DNA-damaging reactive oxygen species and hypochlorous acid [28]. These results collectively show that TNF has a profound effect on the make-up of the stroma during tumour development.

TNF also has a role in neovascularisation, which may have implications for tumour angiogenesis. At low concentrations (0.5–50 ng/mL), TNF induces *in vitro* chemotaxis of bovine adrenal capillary endothelial cells and induces the formation of branching capillary-tube-like structures, but these effects are inhibited at high TNF doses (500 ng/mL) [29]. Lower levels of TNF (0.05–0.5 U/mL) have been shown to stimulate the proliferation of basic fibroblast growth factor-stimulated adrenal cortex-derived capillary endothelial cells, but increased doses (5–50 U/mL) inhibit proliferation in a dose-dependent manner [30]. *In vivo*, TNF also induces capillary blood vessel formation in the rat cornea and the developing chick chorioallantoic membrane at very low concentrations (3.5 and 1 ng, resp.) [29]. Moreover, subcutaneous implantation in mice with a poly-vinyl-alcohol foam disk containing low doses of TNF (0.01–1 ng) induced angiogenesis, whereas at high doses (up to 5 ng) angiogenesis was inhibited [34]. Therefore, TNF can have bimodal, dose-dependent opposing effects within the context of neovascularisation.

The ability of this cytokine to suppress the proliferation of endothelial cells under some conditions renders it as a potential antitumour angiogenesis agent, if strategically delivered to the tumour site. Indeed, a fusion protein composed of mouse TNF and a high affinity antibody fragment to the extradomain B (ED-B) domain of fibronectin, a marker of angiogenesis, induced significant antitumour activity against subcutaneously grown F9 embryonal teratocarcinoma, an effect attributed by the authors to targeting of the tumour vasculature [40]). The activity of this agent was enhanced when used in combination with the chemotherapeutic drug, melphalan; it was suggested that this synergism was in part due to the effects of TNF on the vasculature, including reduction of interstitial pressure and an increase of vascular permeability that ultimately led to enhanced tumour accumulation of melphalan [40, 41]. The use of targeting approaches to facilitate incorporation of TNF into tumour vasculature has also been investigated in preclinical models of PCa, whereby coupling TNF with the CNGRC peptide, a ligand for CD13 which is expressed on tumour vessels, enhanced the therapeutic index of doxorubicin against TRAMP C1 mouse prostate tumours *in vivo* [42].

While TNF has been shown to promote tumour progression through its role in chronic inflammation, it is also important to note that TNF may also directly endow tumour cells with greater metastatic potential. As tumour cells begin to metastasise, they invade and migrate towards endothelial cells in order to enter the bloodstream. This process involves dynamic interactions between selectins expressed on stromal cells for example, endothelial cells, and selectin ligands on cancer cells [43]. TNF has been shown to enhance the *in vitro* migration and invasion of LNCaP cells through increasing the expression of several glycosyl- and sulfo-transferase genes that are involved in the synthesis of mucin-type selectin ligands [31]. As a result, TNF enhanced the binding of LNCaP cells to E, P, and L selectins, which may facilitate the entry of tumour cells into the bloodstream. TNF has also been shown to induce *in vitro* invasion of PC-3 cells; this increased invasion was accompanied by an increase in their expression of matrix metalloproteinase (MMP-)9 and fibronectin, and a decrease in E-Cadherin [32]. These effects were shown to be mediated through the zinc-finger transcriptional repressor, Snail, as Snail siRNA prevented TNF-induced cell invasion [32]. Therefore, it is conceivable that low amounts of TNF within a chronically inflamed tumour microenvironment may help drive tumour cells to undergo epithelial to mesenchymal plasticity and spread to form secondary tumours. As PCa frequently metastasises to bone forming lesions with a predominantly osteoblastic phenotype [44], it is worthwhile to highlight a potential role for TNF in this process. RAW 264.7 pre-osteoclast cells cultured with conditioned media from LNCaP-C4-2B cells prestimulated with recombinant TNF had a 5-fold decrease in gene expression of NF-*κ*B ligand (RANKL), a suppressor of osteoclastogenesis, as compared to LNCaP-C4-2B cells without TNF [45]. Moreover, conditioned media from TNF-stimulated LNCaP-C4-B2 cells [46] induced *in vitro* mineralisation of MC3T3-E1 osteoblast-like cells, suggesting that TNF within the tumour microenvironment could play a role in bone remodelling and promote osteoblastic activity [45].

Despite abundant evidence to suggest that TNF has a role which favours tumour progression, this cytokine was originally identified as a factor that strongly induced tumour necrosis, hence its name. In PCa, TNF has been shown to dose-dependently induce apoptosis of LNCaP cells [35, 36] and also to sensitise these cells to gamma irradiation-induced apoptosis *in vitro* and *in vivo* [47, 48]. However, the finding that LNCaP derived cell lines, C4, C4-2, and C4-2B, all of which are resistant to androgen deprivation are also resistant to TNF [49] suggests that the sensitivity of PCa cells to TNF may play a role in their androgen responsiveness. Supporting this hypothesis is the observation that TRADD expression is significantly lower in these cell lines, and that androgen deprivation actively suppresses TRADD expression in LNCaP cells [49]. While TNF has limited efficacy in directly inducing apoptosis of androgen-independent PCa cell lines such as PC-3 and DU145 [50], TNF still may have therapeutic potential, based on its ability to stimulate antitumour immunity. TNF is a pleiotropic cytokine, which when strategically delivered to tumours locally, may result in therapy-induced inflammation that could be protective against tumours [51]. This is because the net effect of tumour-associated inflammation is dependent on a fine balance between tumour-promoting and tumour-inhibiting actions [51]. TNF may contribute to protective therapy-induced inflammation by inducing the production of other cytokines (e.g., IL-1, IL-6, IL-8, interferon (IFN-)*γ*) and cytotoxic factors (e.g., nitric oxide and reactive oxygen species) by macrophages and NK cells and enhancing the proliferation of T cells alone or synergistically with IL-6 [7, 37]. TNF also promotes the generation of cytotoxic T cells (CTL), and protects them from transforming growth factor (TGF)-*β*-mediated inhibition [38]. Mice deficient in TNF have been shown to be unable to reject syngeneic MC57X fibrosarcomas, but can do so when recombinant TNF is administered [52]. In addition, CTL and NK cells derived from these mice displayed impaired cytotoxicity against tumour cells. In another study, TNF^−/−^ but not wild-type mice failed to recruit NK cells to the peritoneum, the site where a variety of tumour cells including RM1 murine PCa, have been injected [53]. TNF also protects against RM1 PCa-induced apoptosis of dendritic cells, which are antigen presenting cells pivotal in the development of protective antitumour immunity [39]. These results collectively show that TNF can have direct tumour inhibitory effects as well as roles in immune potentiation, which should be exploited in cancer immunotherapies. For these reasons, TNF is often included in protocols of dendritic cell-based cancer vaccines [54].

## 6. Potential Role of TNF in Eicosanoid Pathways in PCa

Eicosanoids, the oxidative metabolites of the essential fatty acid arachidonic acid, are a focus of increasing interest due to a growing body of evidence across broad disciplines that these products may contribute to the development and progression of PCa [55, 56]. The metabolic pathways that control their production have thus become targets for the development of novel agents to treat this disease because these pathways appear to be aberrantly induced in PCa tissues. Progress is limited largely by the preliminary nature of the field and our poor understanding of the quantitative biochemistry of this complex pathway in PCa. There are over 100 eicosanoid products known in humans, many of which are biologically active and their production and metabolism is controlled by at least 40 enzymes with some products being formed by nonenzymatic-oxidative reactions [57, 58].

Given this complexity and lack of knowledge, a key strategy has been to identify and target factors that regulate the pathway, rather than individual biosynthetic enzymes, in the hope that blockade of these regulators will have a broader benefit. Arachidonic acid flux to the eicosanoid pathways in PCa tissues, as in other tissues, appears to be mediated primarily by one of the 20 known mammalian phospholipase A_2_ enzymes, cPLA_2_-*α* (Group IVA PLA_2_). cPLA_2_-*α* is expressed in PCa and pharmacological inhibition of this enzyme results in reduction of tumour size in a PCa xenograft model [59]. This intracellular enzyme is activated by external receptor mediated signals that mobilise calcium or phosphorylate mitogen and/or stress activated kinase pathways, in particular the ERK, p38, and JNK. In chronic inflammatory conditions, the proinflammatory cytokines, including TNF, are important activators of this enzyme and blockade of these cytokines effectively limits the flux of arachidonic acid to the pathway [60].

A second important regulatory feature of the eicosanoid pathway is that, in addition to a cell-type and tissue-specific complement of constitutively present biosynthetic pathway enzymes that define the range of eicosanoid products made under normal physiological conditions, key pathway enzymes have duplicated genes whose expression is responsive to stress signals such as cytokine activation. Induction of these genes results in a rapid increase in the capacity of the eicosanoid pathways to metabolise arachidonic acid. Several of these genes, notably a secreted phospholipase A_2_ (Group IIA PLA_2_, hGIIA, sPLA_2_-IIA) [55] and cyclooxygenase-2 (COX-2) [61], are aberrantly overexpressed at defined stages of PCa progression. In cultured cells and in tissues, inflammatory cytokines, including TNF, are potent inducers of this gene expression, again through activation of mitogen activated protein kinase and NF-*κ*B signalling pathways.

There is growing evidence that inappropriate expression of inflammatory cytokines including TNF may contribute to the aberrant regulation of eicosanoid pathways in PCa. TNF transiently induces steady-state COX-2 protein and mRNA levels over constitutively high basal levels (prostate tissue has unusually high levels of COX-2 relative to other human tissues) in normal prostate epithelial cells [33]. This increase correlates with increased prostaglandin E2 (PGE2) production and reduced apoptosis. In PCa cells, COX-2 is barely detectable without stimulation, but is inducible by TNF. Importantly, the time course of induction [33] and the quantitative production of PGE2 [62] on TNF stimulation is variable between cell lines indicating that TNF signalling is significantly and variably altered, but not ablated in these cells. TNF stimulation of tumour cells may thus directly induce aberrant PGE2 production, affecting downstream regulation of proliferation and apoptosis by PGE2. Further, increased TNF production by surrounding normal cells due to inappropriate TNF expression in the prostate may contribute to increased paracrine PGE2 production, thereby indirectly suppressing apoptosis in cancer cells through PGE2 signalling [33].

Induction of cytokine gene expression, including TNF, can be demonstrated in cultured PC-3 cells following addition of arachidonic acid [63]. This induction is suppressed by pharmacological blockade of PI3 kinase or COX enzymes and correlates with increased phosphorylation of the AKT, without phosphorylation of the MAP kinase pathways ERK, p38, or JNK. Thus TNF stimulation of the eicosanoid pathway may serve to indirectly activate other growth stimulatory signalling pathways and to further amplify cytokine production, even in the absence of immune cell infiltration.

In combination, these data provide evidence that TNF may contribute to PCa growth by directly and indirectly modulating pathways that stimulate proliferation and reduce apoptosis of cancer cells. Studies aimed at blockade of TNF *in vivo* appear warranted in an effort to determine the relative importance of this cytokine over other factors such as stimulators of the HER/HER2 pathway [64] that may activate eicosanoid-related growth stimulatory pathways in PCa.

## 7. A Perspective on TNF Therapy

Much of the confusion relating to the function of TNF in cancer can be attributed to the dependence of its effects on the biological context within which the cytokine has been investigated. Variables such as cytokine dose, target cell type, hormone sensitivity of the cell type, and the complexity of the system (*in vitro* versus *in vivo*) can greatly influence the type of activity seen to be exerted by TNF. By virtue of its dual role in tumour biology, it may appear at face value that TNF would have limited therapeutic utility against cancers because its protumour properties would nullify its antitumour effects. However, here we propose two TNF-centred therapeutic approaches which are rational for the treatment of PCa. The first approach is to neutralise TNF in patients with androgen-sensitive non-metastatic disease. Since TNF is central in chronic inflammation (important in tumour initiation and progression), drives epithelial to mesenchymal plasticity (facilitating metastasis), is involved in the progression of prostate tumours from an androgen-sensitive to CRPC, and may contribute to the aberrant regulation of eicosanoid pathways (stimulate proliferation and reduce apoptosis), then blockade of TNF could keep PCa progression in check. In addition, since elevated serum levels of TNF correlate with increased likelihood of cancer-related cachexia and coagulopathy, neutralisation of TNF may also have palliative effects. A recent pilot study provided anecdotal evidence that neutralisation of TNF may benefit some patients with advanced PCa [65]. Transient pain relief from bone metastases was noted in 2 of 6 patients who received the TNF-blocking antibody, infliximab. While disease progressed in all patients, no treatment-related adverse events were noted. It is important to note that TNF also contributes to Rheumatoid Arthritis by fuelling chronic inflammation, inducing angiogenic factors, modulating the expression of adhesion molecules, and enhancing production of MMPs, and that TNF antagonists have had tremendous clinical success for the treatment of this disease [66]. All of these processes are also implicated in tumour-associated chronic inflammation; therefore, it is conceivable that blockade of TNF could be efficacious against early-stage PCa.

The second therapeutic strategy we propose is targeted-delivery of TNF to the tumour site. The rationale is based on the direct effects of TNF in destroying the tumour vasculature at high doses, and on its effects in stimulating antitumour immunity. Locally delivered high dose TNF in combination with melphalan is already a well-established treatment protocol for soft tissue sarcoma (STS), and melanoma in-transit metastases confined to the limb [4]. In this setting, the ability of TNF to modulate the tumour vasculature has been exploited, allowing for greater accumulation of the chemotherapeutic drug within the tumour. As TNF shows a broad spectrum of effects on immune cells, it also has potential to be used as an immunotherapy for PCa. The rationale is to administer sufficient doses of TNF to the tumour to induce acute inflammation, which frequently precedes the development of adaptive antitumour immunity [51]. In a clinical trial involving 10 patients with locally advanced hormone-resistant PCa, intratumoural injection with recombinant TNF at 4-week intervals combined with intermittent subcutaneous injection of IFN-*α*2b, induced a significant reduction in prostate volume in 9 patients [67]. Tumour necrosis was found in biopsy samples from all patients, some of which contained a heavy infiltration of macrophages and NK cells, indicating local cytotoxic effects by the cytokine [67]. The rationale for delivering TNF to the tumour site as a therapeutic approach is further supported by a clinical trial showing no clinical activity by TNF when administered systemically daily for 5 consecutive days in patients with androgen-independent PCa [68]. In addition, severe dose-limiting toxicities were observed. Methods of cytokine delivery that warrant investigation include intratumoural injection of TNF, conjugation of TNF-coated/carrying nanoparticles to antibodies specific for prostate antigens, and gene therapy approaches whereby expression of this cytokine is driven by prostate-specific promoters. Further support for this therapeutic approach comes from the strong antitumour effects achieved through tumour expression/local delivery of other proinflammatory cytokines including IL-2 [69], granulocyte macrophage-colony stimulating factor (GM-CSF) [70], and IL-18 alone [71] or a combination with IL-12 [72] which have been reported in preclinical *in vivo* models of PCa. Based on the integral role of TNF in promoting and inhibiting PCa growth, TNF is a potential biomarker for the disease and research into its therapeutic utility needs to continue.

## Figures and Tables

**Table 1:** Summary of potential protumour and antitumour roles of TNF in PCaPCa.

Protumour	Reference
Involvement in the initiation of castrate resistant PCa by inducing hypersensitivity to androgen (LNCaP cells)	[12]
Induces neutrophil production of myeloperoxidase, which generates carcinogenic reactive oxygen species (ROS) and hypochlorous acid	[27]
Induces *in vitro *chemotaxis and proliferation of endothelial cells at low doses	[29, 30]
Upregulates E-, P-, and L-selectin ligands on LNCaP cells, which may facilitate extravasation to bloodstream	[31]
Increases expression of MMP-9, fibronectin and decreases E-Cadherin by PC-3 cells	[32]
Involvement in epithelial-mesenchymal plasticity via Snail	[32]
May stimulate tumour proliferation and reduce apoptosis via PGE2	[33]

Antitumour	Reference

Induces regression of normal prostate	[22]
Inhibits *in vitro* and *in vivo* angiogenesis at high doses	[30, 34]
Induces apoptosis of LNCaP cells	[35, 36]
Stimulates antitumour immunity by enhancing the generation and proliferation of cytotoxic T cells (CTL) Also prevents TGF-*β*-mediated inhibition of CTL generation	[7, 37, 38]
Induces production of other cytokines (e.g., IL-1, IL-6, IL-8, and IFN-*γ*) and cytotoxic factors (e.g., NO and ROS) by macrophages and NK cells	[7]
Protects dendritic cells from tumour-induced apoptosis	[39]
